# miR-27b inhibits fibroblast activation via targeting TGFβ signaling pathway

**DOI:** 10.1186/s12860-016-0123-7

**Published:** 2017-01-17

**Authors:** Xiangming Zeng, Chaoqun Huang, Lakmini Senavirathna, Pengcheng Wang, Lin Liu

**Affiliations:** 1 Department of Immunology and Microbiology, Medical School of Jinan University, Guangdong, China; 2 Lundberg-Kienlen Lung Biology and Toxicology Laboratory, Department of Physiological Sciences, Stillwater, OK USA; 3 Oklahoma Center for Respiratory and Infectious Diseases, Oklahoma State University, Stillwater, OK USA

**Keywords:** miR-27b, Fibroblast activation, TGFβ, Idiopathic pulmonary fibrosis

## Abstract

**Background:**

MicroRNAs are a group of small RNAs that regulate gene expression at the posttranscriptional level. They regulate almost every aspect of cellular processes. In this study, we investigated whether miR-27b regulates pulmonary fibroblast activation.

**Results:**

We found that miR-27b was down-regulated in fibrotic lungs and fibroblasts from an experimental mouse model of pulmonary fibrosis. The overexpression of miR-27b with a lentiviral vector inhibited TGFβ1-stimulated mRNA expression of collagens (COL1A1, COL3A1, and COL4A1) and alpha-smooth muscle actin, and protein expression of Col3A1 and alpha-smooth muscle actin in LL29 human pulmonary fibroblasts. miR-27b also reduced contractile activity of LL29. TGFβ receptor 1 and SMAD2 were identified as the targets of miR-27b by 3’-untranslated region luciferase reporter and western blotting assays.

**Conclusions:**

Our results suggest that miR-27b is an anti-fibrotic microRNA that inhibits fibroblast activation by targeting TGFβ receptor 1 and SMAD2. This discovery may provide new targets for therapeutic interventions of idiopathic pulmonary fibrosis.

## Background

Idiopathic pulmonary fibrosis (IPF) is a chronic, progressive, and usually fatal disease. The features of the disease are characterized by repeated injury and activation of alveolar epithelial cells, formation of myofibroblasts and excessive accumulation of extracellular matrix in the lung parenchyma [1]. The estimated prevalence of IPF in the United States is 14 to 27.9 per 100,000 people [2]. However, the etiology and molecular mechanisms of IPF initiation and progression are largely unclear.

Chronic lung inflammation had been considered preceding pulmonary fibrosis and played a major role in lung fibrogenesis. However, some evidence suggested that inflammation was not an important pathogenic event of IPF. For example, in the early or late course of IPF, including diseases similar to mild or moderate alveolitis, there was a lack of long-term effective response to anti-inflammation therapy [3]. Fibroblasts are the main source of abnormal extracellular matrix production in IPF. The TGFβ signaling is an important mediator of pulmonary fibrosis in both animals and humans. TGFβ modulates the activation of fibroblasts [1]. Alveolar epithelial cells also play an important role in the process of pathogenesis of IPF. The activated cells secrete soluble protein factors, including TGFβ, TNFα, endothelin-1 to exert their effects on fibroblasts [1].

MicroRNAs (miRNAs) are a group of endogenous non-coding small RNAs. More than 3000 mature miRNAs are found from plants to humans. miRNAs are the key regulators of many biological processes, and they function by inhibition of translation and degradation of target mRNAs to control protein expression in physiological and pathophysiological conditions [4]. miRNAs are involved in almost all aspects of cell physiology, including cell proliferation and differentiation, apoptosis, and diseases [5–8]. Based on computational analysis, the mRNA targets of miRNAs in most mammals are conserved due to selective pressure [9].

miR-27b has been reported to play a role in breast, liver, kidney or other organs. For example, antagomir of miR-27b suppressed cell invasion in human breast cancer cell line, MDA-MB-231, whereas pre-miR-27b stimulated invasion in ZR75 breast cancer cells [10]. miR-27b synergized with anticancer drugs in a defined subgroup of liver and kidney cancer patients [11]. The overexpression or inhibition of miR-27b in HuH7 cells significantly decreased or increased the peroxisome proliferator-activated receptor (PPAR) alpha protein level [12]. miR-27b targets the 3’-untranslated region (3’-UTR) of PPARγ and inhibits its mRNA and protein expression in neuroblastoma cells [13].

Recently, miR-27b was identified as a major miRNA in modulating TGFβ-induced collagen I expression using a miRNA inhibitor library [14]. The inhibition of miR-27b increased COL1A1 expression. However, this study used lung epithelial cancer cell line A549 cells rather than pulmonary fibroblasts. During the course of this work, Cui et al. published a study showing that transfection of miR-27a-3p mimic into a fetal lung fibroblast cell line MRC-5 inhibited COL1A2 and alpha-smooth muscle actin (α-SMA) and this result was confirmed in IPF fibroblasts [15]. The use of a miRNA mimic could result in overwhelmed expression of a miRNA since it by-pass the cellular regulatory system for the processing of a miRNA.

In this study, we investigated a role of miR-27b in fibroblast activation using a human lung fibroblasts and a lentiviral vector expressing a primary miR-27b, which is converted into a mature miRNA via the endogenous processing system after entering cells. We found that miR-27b inhibited fibroblast activation, and TGFβ receptor 1 (TGFBR1) and SMAD2 are direct targets of miR-27b. Our results suggest that miR-27b is an anti-fibrotic miRNA in pulmonary fibroblasts.

## Methods

### Bleomycin mouse fibrosis model and isolation of primary cells

The animal procedures were approved by the Institutional Animal Care and Use Committee at the Oklahoma State University (VM-15–38). Bleomycin or saline was delivered to the lungs of C57BL/6 male mice (6–8 weeks) via nasal instillation at a dose of 3 U/kg body weight. On day 14 mice were sacrificed, and lung tissues were collected. Fibroblasts and alveolar epithelial type II cells (AEC II) were isolated from the lungs of saline or bleomycin-treated mice according to the previously described protocols [16, 17]. Alveolar epithelial type I cells (AEC I) were obtained by culturing AEC II in Dulbecco’s Modified Eagle Medium (DMEM) for 5 days [18].

### RNA isolation

Total RNAs were isolated from lung tissues or cells by using Tri Reagents (Molecular Research Center, Cincinnati, OH). The RNA concentration and quality were determined by NanoDrop ND-1000 Spectrophotometer.

### Construction of vectors

The primary hsa-miR-27b was PCR-amplified from human genomic DNA (Promega, Madison, WI) with the following primers: forward,TTTCTCGAGGGATTACCACGCAACCAC and reverse, TTTGAATTCGGCTAGCATTCCCAGCAGGAGA. The PCR product was inserted into a modified lentiviral vector pLVX (Clontech, Mountain View, CA) the downstream of its green fluorescent protein (GFP) at XhoI and EcoRI as described [19].

The 3’-UTRs of human TGFβ receptor 1 (TGFBR1) and SMAD2 were PCR-amplified from human genomic DNA with the following primers: TGFBR1 forward, GCTAGCTGAATATTCTCACATCAAGCTTT and reverse, GTCGACGTGAGAAATCATGTATTACAACT.

SMAD2 forward, GCTAGCTTTCTCTAGTGATATTAAGGAACG and reverse, GTCGACACAGATGATGCACACAAATATAT. TGFBR1-UTR or SMAD2-UTR was inserted into the pmirGlo vector (Promega) the downstream of firefly luciferase gene at Nhe I and Sac I. All of the constructs were confirmed by DNA sequencing.

### Preparation of lentivirus

To produce lentivirus overexpressing miR-27b, lenti-miR-27b or its control plasmid was transfected to HEK 293 T cells along with Lenti-X HTX Packaging mix (Clontech) by using Lipofectamine 2000. After a 48-h transfection, the media containing viruses were collected. For virus titer determination, HEK 293 T cells were split into a 12-well plate at a density of 5 × 10^5^cells per well. Cells were infected with lentiviruses at a series of dilutions. 48 h post infection, virus titer was determined by counting GFP-positive cells (10 fields per well) under a fluorescence microscope.

### Infection of fibroblasts with a lentiviral miR-27b

LL29 lung fibroblasts were purchased from American Type Culture Collection (ATCC, Manassa, VA, CCL-134) and were maintained in F12K medium supplemented with 10% fetal bovine serum (FBS) and 1% penicillin/streptomycin (P/S). The fibroblasts were seeded on 6-well plates at a density of 2–5 × 10^5^/well. After 24 h, cells were infected with a lentiviral miR-27b or its control at a multiplicity of infection (MOI) of 50. 48 h post infection, cells were stimulated with TGFβ (5 ng/ml). After another 48 or 72 h, cells were collected for RNA and protein analyses.

### Real-time PCR

Total RNA was reverse-transcribed into cDNA using Moloney Murine Leukemia Virus reverse transcriptase. For miRNA quantitation, total RNA was poly (A)-tailed using an A-Plus Poly (A) Polymerase Tailing Kit (Epicentre, Madison, WI) before reverse transcription. The following primers were used: COL1A1, Forward: CGAAGACATCCCACCAATCAC, and reverse: CAGATCACGTCATCGCACAAC; COL3A1, Forward: TGGCTACTTCTCGCTCTGCTT, and reverse: TTCCAGACATCTCTATCCGCATAG; COL4A1 forward: CTCTGGCTGTGGCAAATGTG, and reverse: CCTCAGGTCCTTGCATTCCA; α-SMA, forward: GTGTTGCCCCTGAAGAGCAT, and reverse: CGCCTGGATAGCCACATACAT; microRNA-universe reverse primer: GCGAGCACAGAATTAATACGAC; RNU6 forward primer: AGAGAAGATTAGCATGGCCCCT; miR-27b forward primer: TTCACAGTGGCTAAGTTCTGC. Real-time PCR was performed using SYBR Green master mix on an ABI 7500 fast system (Applied Biosystems, Foster City, CA). The thermal temperature were: 95°C for 10 min, followed by 40 cycles of 95°C for 15 s, 60°C for 30 s, and 65°C for 30 s. The endogenous reference genes were glyceraldehyde-3-phosphate dehydrogenase (GAPDH) or RNU6. The comparative ΔCt method was used to calculate the relative mRNA and miRNA expression levels.

### Luciferase reporter assay

miR-27b or its control plasmid (150 ng) were co-transfected into HEK 293 T cells using Lipofectamine 2000 with TGFBR1-UTR or SMAD2-UTR plasmid (5 ng), which contains a *Renilla* luciferase gene for normalization. After a 48-h transfection, the cells were harvested, and luciferase activities were measured using the Dual Luciferase Reporter Assay System (Promega).

### Western blot

Proteins (10–20 μg) were separated on SDS-PAGE and transferred onto nitrocellulose membranes. After being incubated with primary antibodies, the membranes were washed with Tris-buffered saline (pH 7.5) and Tween 20 and incubated with horseradish peroxidase-conjugated anti-mouse or rabbit secondary antibodies for 1 h. The target proteins were visualized with Super Signal West Pico Chemiluminescents Substrate and analyzed with Amersham 600 Molecular Imager. The following antibodies and dilutions were used: mouse anti-α-SMA monoclonal antibody (1:1000; Sigma), rabbit anti-β-actin monoclonal antibody (1:1000; Santa Cruz), rabbit anti-TGFBR1 polyclonal antibody (1:500; Santa Cruz), rabbit anti-SMAD2 polyclonal antibody (1:500; Santa Cruz), rabbit anti-Col3A1 polyclonal antibody (1:500; Santa Cruz), goat anti-rabbit monoclonal second antibody (1:2000; Sigma), and goat anti-mouse monoclonal second antibody (1:2000; Sigma).

### Fibroblast contraction assay

LL29 fibroblasts were seeded in 6-well plates at a density of 1–2 × 10^5^ cells per well overnight and infected with miR-27b or its control lentivirus at a MOI of 50 for 48 h. The cells were treated with TGFβ1 (5 ng/ml) for another 48 h. The cells were trypsinized and mixed with rat tail collagen I (BD Bioscience, Cat# 354236) to a final concentration of 1 × 10^5^cells/ml and 1 mg/ml of collagen 1, followed by the addition of 15 μl 0.5 M NaOH to 1 ml of the cells. The cells were then added to 24-well BSA-coated plates (500 μl/well). After a 30-min incubation, medium with or without TGFβ (5 ng/ml) were added (500 μl/well). Cells were incubated for 48 h and images were taken. The gel areas were quantified using Image J software.

### Statistics

All experiments were performed with at least three independent replicates. For statistical analysis, student *t*-test was used for two group comparisons and ANOVA, followed by Turkey’s test for multiple group comparisons. P < 0.05 was considered significance.

## Results

### miR-27b is down-regulated in the lungs and fibroblasts from bleomycin-treated mice

**Fig. 1:**
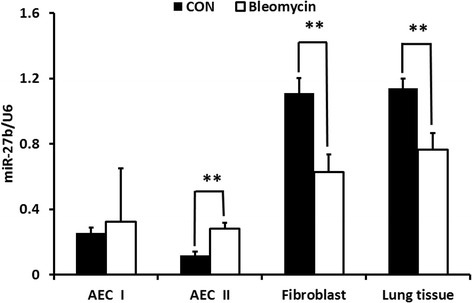
miR-27b expression in the lung tissues and fibroblasts from bleomycin-treated mice. The lung tissues were collected from saline control (*CON*) and bleomycin-treated mice. Fibroblasts and alveolar epithelial type II cells (*AEC II*) were isolated from the lungs. Alveolar epithelial type I cells (*AEC I*) were obtained via trans-differentiation by culturing AEC II for 5 days in vitro. The expression of miR-27b was determined by real-time PCR and normalized to RNU6 (U6). Data shown are means ± S.E. **P < 0.01. *n* = 9 for lung tissues; *n* = 10 for fibroblasts; and *n* = 3 for AEC I and AEC II. Student *t*-test

We determined miR-27b expression levels in the fibrotic lungs induced by bleomycin in mice by real-time PCR. The expression of miR-27b in the lung tissue of bleomycin-treated mice was decreased significantly compared to that of the control mice (Fig. 1). To examine which types of the lung cells account for the decrease, we isolated fibroblasts and alveolar epithelial cells from the bleomycin-treat mice. The expression level of miR-27b in fibroblasts was much higher than alveolar epithelial type I and type II cells (AEC I and AEC II). Bleomycin treatment reduced miR-27b expression in the fibroblasts (Fig. 1), suggesting that fibroblasts are the cells responsible for the reduction of miR-27b in the fibrotic lungs.

### miR-27b inhibits pulmonary fibroblast activation

**Fig. 2:**
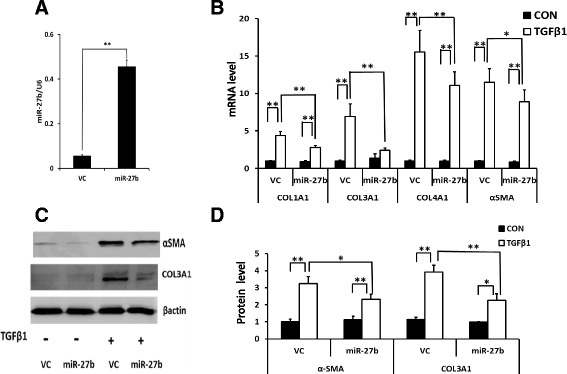
Effect of miR-27b on fibroblast activation. LL29 fibroblasts were infected with a miR-27b lentivirus or virus control (*VC*) at a MOI of 50. **a** miR-27b expression and **b** mRNA expression of COL1A1, COL3A1, COL4A1, and α-SMA were determined by real-time PCR. The expression levels of miR-27b and mRNA were normalized to U6 and GAPDH, respectively. mRNA levels were expressed as fold changes over the control group without TGFβ1 treatment (*CON*). **c**, **d** The protein level expression of α-SMA and COL3A1 were determined by western blotting and quantitated using Image J software. The results were normalized to β-actin and expressed as fold changes over control without TGFβ1 treatment (*CON*). Data shown are means ± S.E, *P < 0.05, **P < 0.01. *n* = 3 for **a** and **d**, *n* = 6–10 for **b**. Student *t*-test for **a** and ANOVA, followed by Turkey’s test for **b** and **d**

To study whether miR-27b affects fibroblasts’ function, we overexpressed miR-27b in LL29 human pulmonary fibroblasts using a lentiviral vector and determined the effect of miR-27b on TGFβ1-mediated collagen and α-SMA expression by real-time PCR and western blotting. The overexpression of miR-27b in LL29 fibroblasts were confirmed (Fig. 2a). The mRNA expression of *COL1A1*, *COL3A1*, *COL4A1* and *α-SMA* was increased by TGFβ1 treatment, and this increase was suppressed by miR-27b overexpression (Fig. 2b). Moreover, the TGFβ1-induced protein expression of Col3A1 and α-SMA was also inhibited by miR-27b overexpression (Fig. 2c, d). These results indicate that miR-27b represses fibroblast activation.

### miR-27b attenuates the contractile activity in pulmonary fibroblasts

**Fig. 3:**
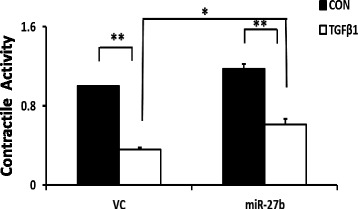
Effects of miR-27b on contractile activity of lung fibroblasts. LL29 lung fibroblasts were infected with miR-27b lentivirus or virus control (*VC*), and then treated with TGFβ1. The cells were mixed with collagen I and seeded in 24-well plates for 48 h. Images were taken and gel areas were quantified using Image J. Collagen gel contractile activity was calculated as gel surface area divided by well surface area. The results are expressed as a fold change over VC without TGFβ1 treatment (*CON*). Data shown are means ± S.E., *n* = 5,*P < 0.05, **P < 0.01. ANOVA, followed by Turkey’s test

The contraction process is known to be mediated by specialized fibroblasts in IPF and TGFβ signaling pathway regulates this process. Therefore, we investigated whether miR-27b influences the contractility of pulmonary fibroblasts. As shown in Fig. 3, miR-27b inhibited the TGFβ1-induced contractility of LL29 fibroblasts.

### miR-27b directly targets TGFBR1 and SMAD2

**Fig. 4:**
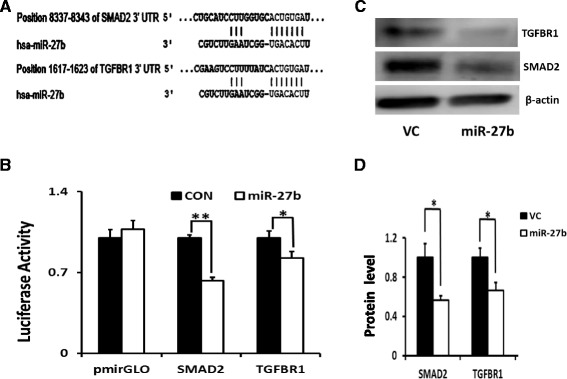
TGFBR1 and SMAD2 are the targets of miR-27b. **a** The binding sites of miR-27b on the 3’-UTRs of TGFBR1 and SMAD2 as predicted by TargetScan. **b** 3’-UTR luciferase reporter assay. HEK 293 T cells were transfected with TGFBR1-UTR or SMAD2-UTR luciferase vector together with the miR-27b expression plasmid or vector control (CON). pmiRGLO is the empty vector without any 3’-UTR sequences. Luciferase activities were determined 48 h post-transfection. The fold changes were relative to CON for each reporter construct. Data shown are means ± S.E *P < 0.05, **P < 0.01 *n* = 4. **c**, **d** TGFBR1 and SMAD2 protein levels in miR-27b overexpressing fibroblasts. LL29 fibroblasts were infected with a miR-27b lentivirus or virus control (VC) at a MOI of 50. After 72 h, the cells were lysed for Western blotting. Protein levels were quantitated by Image J software and normalized to β-actin. The fold changes were relative to VC. Date were represented as means ± S.E., *P < 0.05, *n* = 3–4. ANOVA, followed by Turkey’s test

Because miR-27b inhibits TGFβ1-induced fibroblast activation, it likely targets the components in the TGFβ signaling pathway. Using TargetScan, TGFBR1 and SMAD2 were identified as the potential targets of miR-27b (Fig. 4a). Next we constructed 3’-UTR luciferase reporter vectors and performed the dual luciferase assay to validate whether TGFBR1 and SMAD2 are the direct targets of miR-27b. As shown in Fig. 4b, miR-27b significantly inhibited the luciferase activities of TGFBR1-UTR and SAMD2-UTR reporters. Furthermore, TGFBR1 and SMAD2 protein expression was inhibited by miR-27b in LL29 fibroblasts (Fig. 4c, d).

## Discussion

miRNAs play important roles in various biological processes, such as cell proliferation, differentiation, apoptosis, and other functions. miRNAs show different expression profiles in many diseases including IPF. Abnormal expression of miRNAs may contribute to the development of many diseases. miRNAs may be potentially utilized in the treatment of human diseases, such as infectious and metabolic diseases [20, 21]. In the present study, we demonstrated that miR-27b was down-regulated in fibrotic lungs and fibroblasts from bleomycin-induced mouse pulmonary fibrosis model. In contrast, miR-27a-3p was found to be increased in the fibroblasts isolated from the lungs of the bleomycin-treated mice although the same authors found that miR-27a-3p was decreased in IPF fibroblasts compared to normal lung fibroblasts [15]. Additionally, TGFβ inhibited miR-27b expression in lung epithelial A549 cells [14], but increased miR-27a expression in MRC-5 fibroblasts [15]. The reasons for these differences remain to be determined, but could be due to the differences in transcription because miR-27a-3p and miR-27b are located in different chromosomes, chromosome19 and chromosome 9, respectively. However, there is only one base difference between miR-27a-3p and miR-27b.

The abnormal activation of fibroblasts is one of the major factors driving fibrotic progression in IPF [22–24]. TGFβ activates fibroblasts and enhances collagen synthesis and extracellular matrix deposition [25–27]. In the current study, we found that ectopic overexpression of miR-27b suppressed TGFβ-induced fibroblast activation, as evidenced by the decreased collagen synthesis, inhibition of α-SMA mRNA and protein expression, and enhanced contractile ability. A recent report shows that miR-27b inhibitor increased TGFβ-induced COL1A1 expression in lung epithelial A549 cells [14]. The significance of this regulation in lung epithelial cells is unclear since pulmonary fibroblasts are the major cells for extracellular matrix deposition in IPF. Most recently, miR-27a-3p has been shown to inhibit TGFβ-induced COL1A2 and α-SMA expression and gel contractility in MRC-5 fibroblasts [15]. However, a miR-27b-3p mimic was used for these studies, which may result in overwhelmed expression of miR-27a. Further, MRC-5 fibroblasts are derived from fetal lungs, which may not be the best fibroblast cell line for studying IPF, which normally occurs in patients between the ages of 50 and 70 years. Whether miR-27a-3p and miR-27b have the same functions or are redundant in pulmonary fibroblasts needs further investigations.

Several miRNAs are involved in the regulation of pulmonary fibrosis through the TGFβ/SMAD pathway, including miR-21, miR-26a and miR-29 [28–30]. We suspected that miR-27b may act in a similar mechanism in pulmonary fibrosis. Indeed, TGFBR1 and SMAD2 were identified as potential targets of miR-27b by TargetScan. The dual-luciferase reporter assay confirmed that miR-27b functioned through a direct binding to its 3-’UTR of TGFBR1 and SMAD2. The endogenous protein expressions of these targets were reduced in LL29 fibroblasts by miR-27b, which further confirmed that TGFBR1 and SMAD2 are the targets of miR-27b. Using similar approaches, Cui et al. found that miR-27a-3p targeted SMAD2/4 in fetal lung MRC-5 fibroblasts and they also identified α-SMA as an additional target of miR-27a-3p [15]. In lung epithelial A549 cells, miR-27b targets gremlin 1 [14]. In neuroblastoma cells, PPARγ is a target of miR-27b as determined by 3’-UTR luciferase reporter and endogenous protein assays [13]. In liver cells, miR-27b regulates PPARα indirectly since overexpression of miR-27 reduced the PPARα protein level, but 3’-UTR luciferase reporter assay did not confirm PPARα as a direct target protein level [12]. It appears that miR-27a-3p and miR-27b have multiple targets and which genes are the main targets may depend on cell types.

## Conclusions

To sum up, our present studies show that miR-27b plays an important role in the pulmonary fibroblast activation by regulating TGFBR1 and SMAD2.

## Abbreviations

3’-UTR: 3’ Untranslated region
AEC I: Alveolar epithelial type I cells
AEC II: Alveolar epithelial type II cells
COL: Collagens
FBS: Fetal bovine serum
GAPDH: Glyceraldehyde-3-phosphate dehydrogenase
GFP: Green fluorescent protein
IPF: Idiopathic pulmonary fibrosis
miRNAs: microRNAs
MOI: Multiplicity of infection
P/S: Penicillin/streptomycin
PPAR: Peroxisome proliferator-activated receptor
TGFBR1: TGFβ receptor 1
α-SMA: Alpha-smooth muscle actin
